# Internally labeled Cy3/Cy5 DNA constructs show greatly enhanced photo-stability in single-molecule FRET experiments

**DOI:** 10.1093/nar/gku199

**Published:** 2014-03-15

**Authors:** Wonbae Lee, Peter H. von Hippel, Andrew H. Marcus

**Affiliations:** 1 Oregon Center for Optics and Department of Chemistry, University of Oregon, Eugene, OR 97403, USA; 2 Institute of Molecular Biology and Department of Chemistry, University of Oregon, Eugene, OR 97403, USA

## Abstract

DNA constructs labeled with cyanine fluorescent dyes are important substrates for single-molecule (sm) studies of the functional activity of protein–DNA complexes. We previously studied the local DNA backbone fluctuations of replication fork and primer–template DNA constructs labeled with Cy3/Cy5 donor–acceptor Förster resonance energy transfer (FRET) chromophore pairs and showed that, contrary to dyes linked ‘externally’ to the bases with flexible tethers, direct ‘internal’ (and rigid) insertion of the chromophores into the sugar-phosphate backbones resulted in DNA constructs that could be used to study intrinsic and protein-induced DNA backbone fluctuations by both smFRET and sm Fluorescent Linear Dichroism (smFLD). Here we show that these rigidly inserted Cy3/Cy5 chromophores also exhibit two additional useful properties, showing both high photo-stability and minimal effects on the local thermodynamic stability of the DNA constructs. The increased photo-stability of the internal labels significantly reduces the proportion of false positive smFRET conversion ‘background’ signals, thereby simplifying interpretations of both smFRET and smFLD experiments, while the decreased effects of the internal probes on local thermodynamic stability also make fluctuations sensed by these probes more representative of the unperturbed DNA structure. We suggest that internal probe labeling may be useful in studies of many DNA–protein interaction systems.

## INTRODUCTION

Single-molecule Förster resonance energy transfer (smFRET) is an important technique for the study of local conformations and dynamics of proteins and nucleic acids (1–5). By simultaneously observing changes in fluorescence from donor–acceptor FRET chromophore pairs, it is possible to monitor real-time fluctuations of the relative orientation and separation between site-specifically labeled positions within biomolecular complexes (1,3,6–11). Fluorescent chromophore probes that are well suited to smFRET applications must possess a number of critical properties. These include a high extinction coefficient, a high fluorescence quantum yield, a short triplet state lifetime and a high threshold to photochemical damage (3,12,13). The cyanine dyes Cy3 and Cy5 are currently the most widely used chromophores for smFRET applications, and typical experiments rely on oxygen scavenging and triplet quenching reagents to reduce the effects of photo-induced triplet ‘blinking’ and bleaching (13–18). Nevertheless, chromophore photo-degradation remains a major limiting factor in smFRET experiments that attempt to monitor signals over long observation times (3,12–13,19).

In general, the photo-physical properties of a fluorescent chromophore are sensitive to the dynamics of its local environment (2,20–22). Upon electronic excitation, the induced polarization of the electronic transition can couple to the vibrational motions of the probe molecule itself, in addition to those of nearest-neighbor solvent molecules. Such vibrational motions tend to stabilize the excited electronic state, and they can guide the ensuing relaxation processes. This effect is known as fluorophore ‘solvation’, and it can influence excitation and emission energies, spectral line shapes and fluorescence quantum yields.

Fluorophores that are site-specifically bound to a biological macromolecule are often used to probe local changes in macromolecular conformation by spectroscopic means (1,3,6). In addition to its photo-physical properties, the photo-stability of the chromophore depends on its interactions with the solvent. It is known that small-molecule additives that function as ‘triplet-state-quenchers’ (TSQs), such as β-mercaptoethanol and Trolox, can enhance the photo-stability of cyanine dyes by reducing their triplet state lifetime and dark-state recovery time. Excited triplet states can act as ‘gateways’ to photo-induced degradation pathways, and the activity of TSQ agents seems to be based on a collision-induced solvent accessibility mechanism (12,14–16,23). For example, the photo-stability of duplex DNA constructs labeled with Cy5 can be improved by using a flexible chemical linker to attach a ‘protective’ TSQ group close to the chromophore insertion site (12,24).

**Figure 1.**
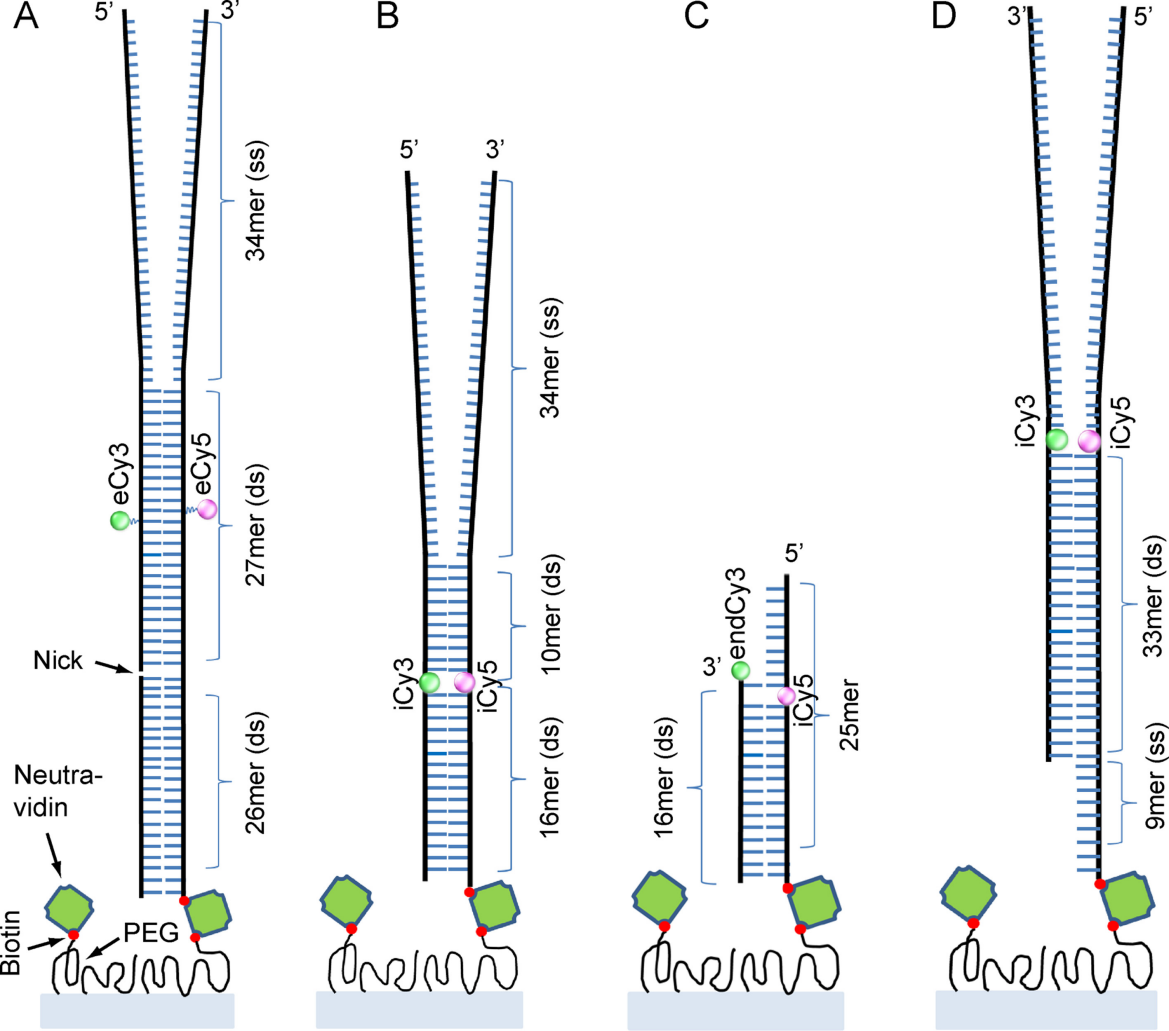
The Cy3/Cy5-labeled DNA constructs used in these studies. (**A**) The Cy3 and Cy5 chromophores are attached to thymine bases on opposite strands by a 16-atom flexible linker. Because the chromophores are positioned externally to the DNA duplex, the substrate is designated ‘eCy3/eCy5 duplex-labeled’ DNA. (**B**) The Cy3 and Cy5 chromophores are rigidly incorporated into the sugar-phosphate DNA backbone using phosphoramidite chemistry. The internally labeled substrate is designated ‘iCy3/iCy5 duplex-labeled’ DNA. (**C**) In this primer/template (p/t) DNA construct, the Cy3 chromophore is attached to the 3’-terminus of the primer strand and the Cy5 chromophore is internally positioned within the template strand near the p/t junction. This substrate is designated ‘endCy3/iCy5 p/t labeled’ DNA. (**D**) The Cy3 and Cy5 chromophores are internally positioned at the single-stranded–double-stranded junction of a DNA replication fork. The substrate is designated ‘iCy3/iCy5 fork-labeled’ DNA.

Here we report an alternative approach to achieving enhanced photo-stability with Cy3/Cy5 labeled DNA constructs, which can be readily implemented using standard smFRET labeling and imaging techniques. We have found that DNA constructs that ‘rigidify’ the excited state of the chromophore—thereby partially decoupling the electronic transition from both intra- and inter-molecular vibrational modes—exhibit favorable photo-physical properties for smFRET experiments. smFRET experiments were used to study the photo-stability of four different Cy3/Cy5 DNA constructs (see Figure 1 and Supplementary Table S1 for replication fork structures and labeling) to investigate the dependence of photo-stability on probe position, labeling chemistry and local conformational stability of the DNA. The DNA samples that we studied can be purchased commercially (e.g. from Integrated DNA technologies, Inc.), and can be assembled into forked and primer–template constructs to study the ‘breathing’ properties of the DNA fork construct itself, as well as to form substrates for the study of helicase and polymerase mechanisms and their interactions with their nucleic acid targets.

In single-molecule studies these nucleic acid constructs are typically attached to microscope coverslips using standard biochemical methods, which involve coating the quartz surfaces with functionalized poly(ethyleneglycol) (PEG) and using biotin/neutravidin linkages to attach the constructs to the PEG (3,10,11). The results presented here show that internally labeled probes are much more resistant to photo-damage and exhibit dramatically reduced ‘background’ signal fluctuations relative to probes attached ‘externally’, thus making them useful in smFRET and sm Fluorescent Linear Dichroism (smFLD) experiments that require monitoring DNA backbone motions over lengthy periods of time. In the Discussion, we consider possible mechanistic explanations for the enhanced photo-stability and local thermodynamic stability provided by these internal probes, and suggest how these attributes might make them useful in the study of other macromolecular systems as well.

## MATERIALS AND METHODS

### smFRET experiments

We used a custom built smFRET instrument and sample preparation methods as described elsewhere (11). Briefly, we performed smFRET measurements using a prism-based total internal reflection fluorescence microscope. Fluorescence was imaged using a 100× NA = 1.4 oil-immersion objective (Plan Apo, Nikon) and filtered using a long-pass filter (BLP01–532R-25, Semrock, Rochester, NY). The fluorescence from the Cy3 (donor) and Cy5 (acceptor) chromophores was spatially separated using short beam paths and a minimal number of transmissive optical components. The fluorescence image was directed onto a dichroic mirror (FF650-Di01–25×36, Semrock, Rochester, NY) that was positioned at the input of the beam-separation optics, which was arranged as in a *Sagnac* interferometer. The dichroic mirror produced two counter-propagating light paths (donor and acceptor fluorescence) using two broadband dielectric mirrors (BB1-E02, Thorlabs, Newton, NJ). Each image was projected onto one-half of the active area of an electron-multiplied charge-coupled-device camera (EM-CCD, iXon DV897-BB, 512 × 512 pixels, Andor Technology, Belfast).

Micro-fluidic sample chambers were constructed by pipetting a 1.5 μL drop of standard buffer solution onto a PEG-coated quartz slide. A cover slip (∼10 mm × 24 mm) was placed on top of the slide with the PEG-coated side facing inward and the surfaces were sealed using 5-min epoxy cement. In this way, a sample chamber with a thickness of ∼10 μm could be prepared without inducing spherical aberration, which becomes a problem once the thickness of the flow cell exceeds a few tens of microns when an oil immersion lens is used. Teflon (PTFE) tubing (TT26, 0.914 mm O.D., Weico Wire & Cable, Edgewood, NY) was connected to the pre-drilled holes in the slide and sealed with epoxy. After the sample chamber was dry, a 50 μL aliquot of 0.1–0.2 mg/ml neutravidin (Thermo Scientific, Rockford, IL) was injected into the inlet tube and incubated for 2–3 min before flushing with standard buffer.

Functionalized DNA strands were purchased from Integrated DNA Technologies (Coralville, IA) as shown in Figure 1 (see Supplementary Table S1 for DNA base sequences). The DNA constructs were annealed by heating to ∼90°C for 3–4 min and then allowed to cool slowly to room temperature (∼22°C). The annealed constructs were diluted to a concentration of 50–100 pM, and a 50 μL aliquot was introduced into the sample chamber and incubated for 2–3 min. Unbound DNA molecules were washed away using the standard buffer solution.

All of our single-molecule experiments were conducted using standard imaging buffer (10 mM Tris at pH 8.0, 100 mM NaCl, 6 mM MgCl_2_). These solutions contained the oxygen-scavenging and triplet-quenching system [165 U/ml glucose oxidase (Sigma), 0.8% (w/v) D-glucose (Sigma), 2170 U/ml Catalase (Sigma) in saturated (≥2 mM) Trolox (Sigma)], which has been shown to work well with the Cy3/Cy5 smFRET chromophore system used in our experiments (3,10,11,14). For a typical data-acquisition run, sequential images were recorded at 100-ms intervals for a total duration of 2 min. Individual image frames contained on average ∼400 Cy3 (donor)/Cy5 (acceptor) single-molecule features, which were analyzed using software written in the IDL programming language. These data were used to construct single-molecule time-dependent trajectories for the donor–acceptor chromophore intensities *I_D_* and *I_A_*, permitting calculation of the smFRET efficiency, which is defined as *I_A_*/(*I_D_* + *I_A_*). We designate a precipitous drop in the value of the FRET efficiency, corresponding to a simultaneous decrease in *I_A_* and increase in *I_D_*, as a ‘FRET conversion event.’

The photo-bleach time constant, τ_B_, of the detected number of FRET pairs was determined by fitting the data to a single exponential decay using Origin software (Northampton, MA) (Figure 3, Table 1, and Supplementary Figure S3).

**Figure 2.**
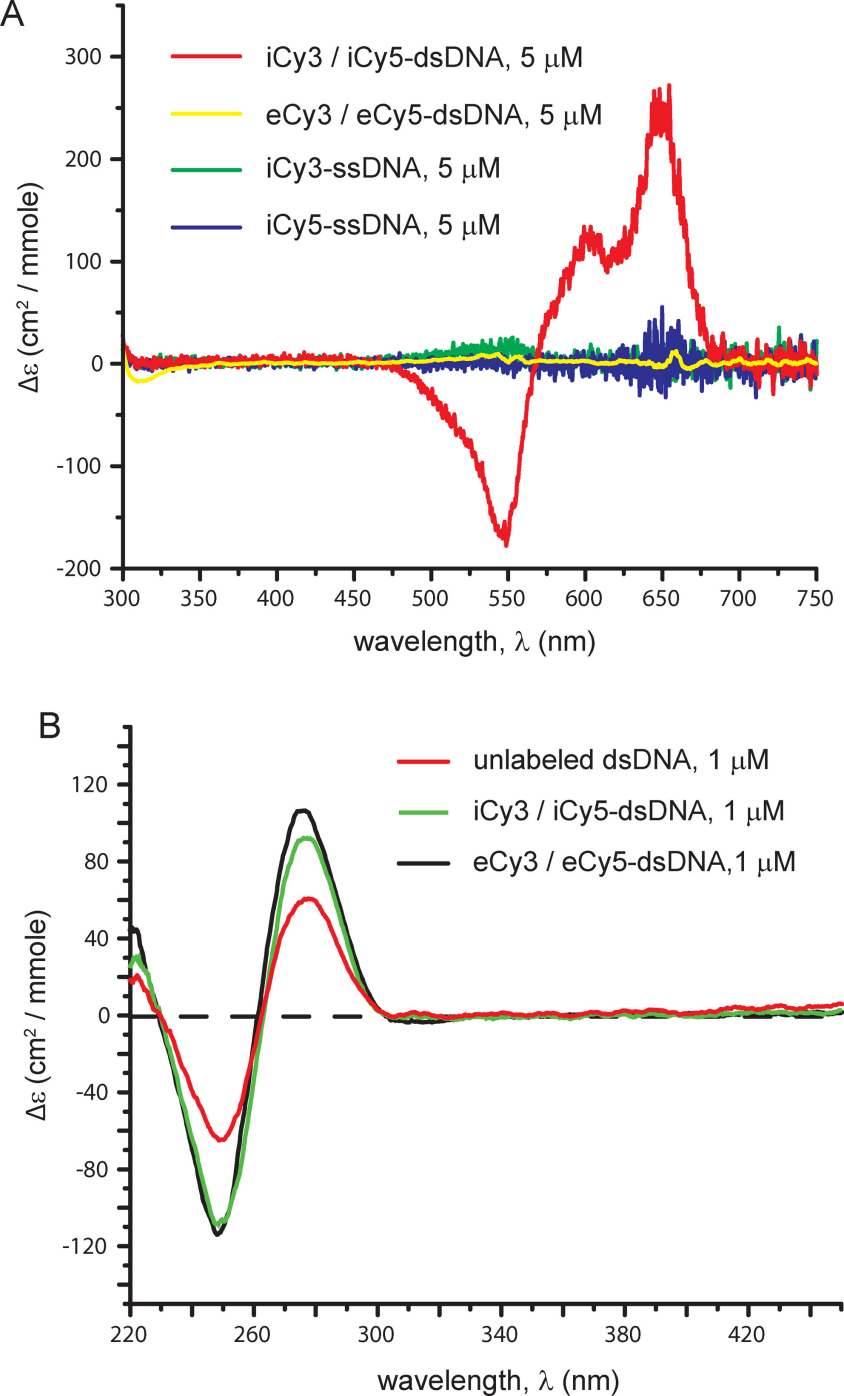
The effect of ‘internal’ versus ‘external’ Cy3/Cy5 labeling on the CD spectra of fully annealed DNA replication fork constructs and the constituent ssDNA strands. (**A**) The iCy3/iCy5-labeled duplex DNA construct exhibits a large CD signal near the probe absorption maximum (red curve), indicating that the chromophores are rigidly fixed within the duplex sugar-phosphate backbone. In contrast, no significant CD signal was observed for the separate iCy3- and iCy5-labeled ssDNA strands (green and blue curves, respectively), nor for the fully annealed eCy3- and eCy5-labeled duplex DNA constructs (yellow curve). All constructs were measured at 5 μM concentrations of DNA molecules. (**B**) The CD spectra of internally and externally labeled Cy3/Cy5-dsDNA constructs at λ < 300 nm and 1 μM concentrations show that the base pairs of the duplex portions of the DNA constructs remain fully stacked in the presence of either the internal or the external Cy3/Cy5 dye probes, and that the presence of either type of probe does not significantly perturb the overall B-form conformation of the duplex regions.

**Figure 3.**
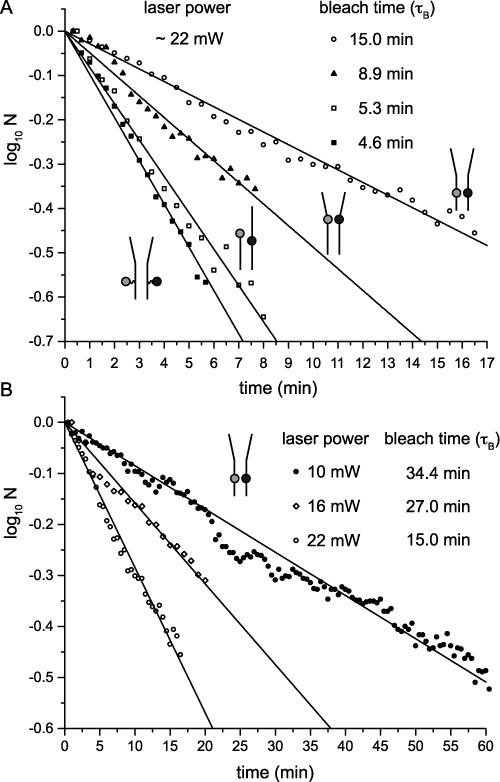
The number of detected Cy3/Cy5 FRET chromophore pairs within a fixed imaging area as a function of time of continuous sample illumination. (**A**) The laser power was set to ∼22 mW. Each of the four DNA constructs exhibited exponential time-dependent photo-degradation, with a lifetime *τ_B_* that depends on chromophore insertion site position and chemistry. Note that the different substrates are identified schematically. (**B**) The results for the ‘iCy3/iCy5 duplex-labeled’ DNA construct are shown as a function of laser excitation power.

**Table 1.** Decay time constants determined from photo-bleach experiments shown in Figure 3 and FRET conversion activity, including partitioning between irreversible and reversible events as shown in Figure 5

log *N* versus time			Slope of best-fit line		S.D.	*R*^2^
eCy3/eCy5 duplex-labeled DNA^a^			−0.0976		0.0015	0.996
iCy3/iCy5 duplex-labeled DNA^a^			−0.0285		0.0003	0.996
endCy3/iCy5 p/t-labeled DNA^a^			−0.0819		0.0015	0.994
iCy3/iCy5 fork-labeled DNA^a^			−0.0488		0.0009	0.991
iCy3/iCy5 duplex-labeled DNA^b^			−0.0158		0.0003	0.993
iCy3/iCy5 duplex-labeled DNA^c^			−0.0085		0.0001	0.994
Single exponential time and rate constants			*τ_B_* (min)		*k_B_* (min^−1^)	*t*_1/2_ (min)
eCy3/eCy5 duplex-labeled DNA^a^			4.6		0.217	3.2
endCy3/iCy5 p/t-labeled DNA^a^			5.3		0.189	3.7
iCy3/iCy5 fork-labeled DNA^a^			8.9		0.112	6.2
iCy3/iCy5 duplex-labeled DNA^a^			15.0		0.066	10.4
iCy3/iCy5 duplex-labeled DNA^b^			27.0		0.037	18.7
iCy3/iCy5 duplex-labeled DNA^c^			34.4		0.029	23.8

FRET conversion analysis	Total FRET (%)	S.D. total FRET (%)	Irreversible FRET (%)	S.D. irrev. FRET (%)	Reversible FRET (%)	S.D. revers. FRET (%)
eCy3/eCy5^a^	37.7	2.81	31.9	3.26	5.8	1.66
iCy3/iCy5 duplex^a^	0.8	0.36	0.5	0.37	0.3	0.35
endCy3/iCy5^a^	17.9	4.55	14.7	3.35	3.2	1.31
iCy3/iCy5 fork^a^	11.9	1.61	9.1	2.49	2.8	0.91

^a^Figure 3A with the laser power at 22 mW.

^b^Figure 3B with the laser power at 16 mW.

^c^Figure 3B with the laser power at 10 mW.

Note: All values were determined by fitting the data with a linear fit and single exponential decay fit using Origin software (OriginLab).

### Thermal denaturation measurements

DNA constructs were assembled and annealed by mixing 1 μM biotinylated leading strand and 1 μM non-biotinylated lagging strand in a 1:1 ratio in various buffers. The solutions were heated to 90°C for 3−4 min and then slowly cooled to room temperature (∼22°C). The annealed constructs were then diluted to a concentration of 500 nM for measuring absorbance at 260 nm using a Cary Model 3E ultraviolet (UV)/visible (VIS) spectrophotometer, which was equipped with a Peltier temperature controller. Samples were heated at 1°C/min from 15°C to 85°C and the absorbance was measured at 1°C intervals.

### Circular dichroism experiments

DNA constructs were assembled and annealed as described in the previous section. The concentrations of the DNA construct for circular dichroism (CD) measurements were 5 μM or 1 μM (Figure 2 and Supplementary Table S1), and the measurements were made at room temperature under a constant flow of nitrogen gas and in standard imaging buffer (10 mM Tris at pH 8.0, 100 mM NaCl and 6 mM MgCl_2_). CD spectra were measured between λ = 200 and 750 nm using a Jasco model J-720 CD spectrometer (Tokyo, Japan), which was equipped with a temperature controlled cell holder. CD spectra were recorded at a scan rate of 100 nm/min.

## RESULTS

### ‘Externally labeled’ DNA constructs

The first sample that we studied was a standard ‘externally labeled’ helicase assay construct involving a model DNA replication fork with the Cy3 and Cy5 chromophores attached to bases on opposing strands using flexible linkers (see Supplementary Table S1 and Figure S1 in Supplementary Information). The labeled bases in this sample are positioned well into the stable duplex region of the construct (Figure 1A). The linker that tethered the external probes to the bases (Supplementary Figure S1) consisted of a 16-atom aliphatic chain, permitting the cyanine dyes to extend beyond the major grooves of the duplex and, in principle, to freely sample a broad range of orientational conformations. The absorbance spectra of the externally labeled dye pairs attached to the DNA constructs at two different construct concentrations are shown in Supplementary Figure S2, and demonstrate that the dyes are present in the constructs and are not perturbed by concentration-dependent interactions between constructs. The absence of a CD spectrum in the visible wavelength region (Figure 2A) indicates that the externally labeled chromophore probes in these constructs are not held in a fixed conformation, but are likely mobile and disordered. At the same time the presence of the normal B-form CD spectrum in the UV region (Figure 2B) confirms that these external probes do not significantly perturb the overall structure of the DNA duplex (25–26).

This external labeling method has generally been used in preparing DNA constructs for single-molecule studies of DNA and DNA–protein interactions, in part to minimize concerns that normal base-pairing and stacking interactions within the constructs might be adversely affected by the presence of the chromophores and also to favor the free rotation of the probes in FRET assays that is often assumed in making Förster energy transfer distance calculations. Specifically, such externally labeled DNA constructs have often been used in single-molecule investigations of helicase-catalyzed nucleic acid unwinding reactions (4–5). Because the probes are bound ‘externally’ to the DNA duplex region, we refer to DNA constructs such as that shown in Figure 1A as ‘eCy3/eCy5 duplex-labeled’.

### ‘Internally labeled' DNA constructs

Our second class of DNA constructs was similar to the first in terms of base composition and sequence, but here the cyanine dyes were rigidly incorporated into the sugar-phosphate backbone as ‘internal’ probes, using phosphoramidite chemistry (Figure 1B) (11,27). Evidence that these internal chromophores are structurally rigid within the helical duplex was provided by the well-developed CD spectra obtained in the visible region with these samples (Figure 2A), as well as by the results of prior studies (10–11). Again CD measurements in the UV confirm that the chromophore probes do not significantly perturb the duplex structure of the DNA constructs (Figure 2B), and unwinding studies showed that these ‘internal’ chromophores do not significantly impede the passage of (at least) the T4 primosome helicase through the replication fork construct (11). Furthermore, the rigid incorporation of the probe chromophores into the sugar-phosphate backbone in these samples was of central importance to experiments that were used to detect ‘breathing’ motions of the DNA backbone on microsecond time scales with a combination of smFRET and fluorescence-detected linear dichroism (smFLD) (10). Because the probe chromophores are bound ‘internally’ within the DNA duplex region, we refer to such samples as ‘iCy3/iCy5 duplex-labeled’ DNA constructs.

### Primer–template and fork-junction DNA constructs

The Cy3/Cy5 chromophores were placed close to a primer/template (p/t) junction (Figure 1C) in a third class of samples. Here the donor Cy3 chromophore was attached to the 3′ end of the primer strand, while the acceptor Cy5 chromophore was incorporated into the sugar-phosphate backbone of the template strand at the ‘+1 position’ relative to the p/t junction (28). We refer to these samples as ‘endCy3/iCy5 p/t junction-labeled’ DNA constructs. In a final class of constructs assembled into model replication forks, the Cy3/Cy5 probes were inserted into the sugar-phosphate backbone at positions close to the single-stranded–double-stranded DNA fork junction (Figure 1D) (10). We refer to these samples as ‘iCy3/iCy5 fork-labeled’ DNA constructs.

### Probe photo-stability as a function of DNA construct environment

We used a prism-type smFRET instrument for our photo-stability characterization studies (11). The various DNA constructs depicted in Figure 1 were dissolved in a standard imaging buffer containing oxygen scavenging and triplet quenching reagents, and were continuously illuminated using a 532 nm laser at power settings of 10–22 mW (3,14). In our single-molecule experiments, the Cy3/Cy5 fluorescence of ∼400 DNA constructs was simultaneously monitored using a split-screen imaging system (11). From analyses of these data, we determined the time required for the Cy3 chromophore to photo-degrade by monitoring changes in the number of single chromophores detected within a fixed imaging area as a function of time.

For all of the above samples, we found that the number of active Cy3 chromophores decreased exponentially over the course of several minutes, with characteristic photo-bleach times, *τ_B_*, that depended on both the labeling chemistry used and the insertion site position within the construct (Figure 3, Supplementary Table S1 and Supplementary figure S1, and Supplementary figures S4–S8). We examined the effects of ‘external’ versus ‘internal’ labeling chemistry on chromophore photo-stability by comparing the results obtained with the eCy3/eCy5 duplex-labeled DNA construct to those obtained with the iCy3/iCy5 duplex-labeled construct. We found that Cy3 photo-degradation occurred nearly three times more rapidly for the externally labeled DNA duplex construct than for the equivalent internally labeled construct. We further examined the effects of changing the probe position and insertion site for the other internally labeled DNA constructs (Figure 1C and D). Figure 3A shows that for the iCy3/iCy5 fork-labeled DNA construct we obtained a photo-bleach time that was significantly reduced in comparison to the iCy3/iCy5 duplex-labeled DNA construct. For the end-Cy3/iCy5 p/t junction-labeled construct, the photo-bleach time was similar to that of the externally labeled DNA construct.

We note that the similarity between the photo-bleach times of the eCy3/eCy5 duplex-labeled DNA construct and the endCy3/iCy5 p/t junction-labeled DNA construct is consistent with the idea that structural rigidity of the probe at the insertion site position might be an underlying factor that controls the photo-stability of these samples. Furthermore, and perhaps contrary to expectations based on the excited-triplet-state collision-induced deactivation model (12,14–15), our results suggest that rigid incorporation of the chromophores into the sugar-phosphate backbone—and the consequent decoupling of the electronic transition from local vibrational motions—may significantly *enhance* chromophore photo-stability. This effect was even more pronounced when the probes were rigidly incorporated into the stable DNA duplex. We tested the dependence of the photo-stability of the probes on the incident laser power for the iCy3/iCy5 duplex-labeled DNA construct (Figure 3B and Supplementary Figure S3) and on the local stability of the DNA duplex. As shown in Figure 3, a reduction in input laser power from 22 mW to 10 mW increased the photo-bleach lifetime significantly. In summary, we emphasize that smFRET signals from the iCy3/iCy5 duplex-labeled DNA construct can be monitored without significant photo-degradation for periods as long as 1 h using continuous illumination with ∼10 mW incident power (Figure 3B and Supplementary Figure S8).

### ‘Reversible’ and ‘irreversible’ FRET conversion events

In smFRET experiments, a FRET conversion event—i.e. a decrease in the Cy5 acceptor signal that is coincident with an increase in the Cy3 donor signal—is often interpreted as a biologically relevant event, such as a macromolecular conformational change, a helicase-driven unwinding process, a protein folding or unfolding event, etc. For instance, under conditions in which the rate of photo-degradation is similar to the rate of an expected conformational change, a Cy5 photo-bleaching event could be mistakenly identified as a conformational transition because both processes give rise to identical changes in the smFRET signal. Nevertheless, a photo-bleaching event is expected to result in an ‘irreversible’ smFRET change, while a local conformational fluctuation is expected to be ‘reversible.’ In order to distinguish between photo-bleaching and conformational fluctuations, we examined the influence of probe labeling chemistry and insertion site position on the rate of spontaneous smFRET conversion events.

**Figure 4.**
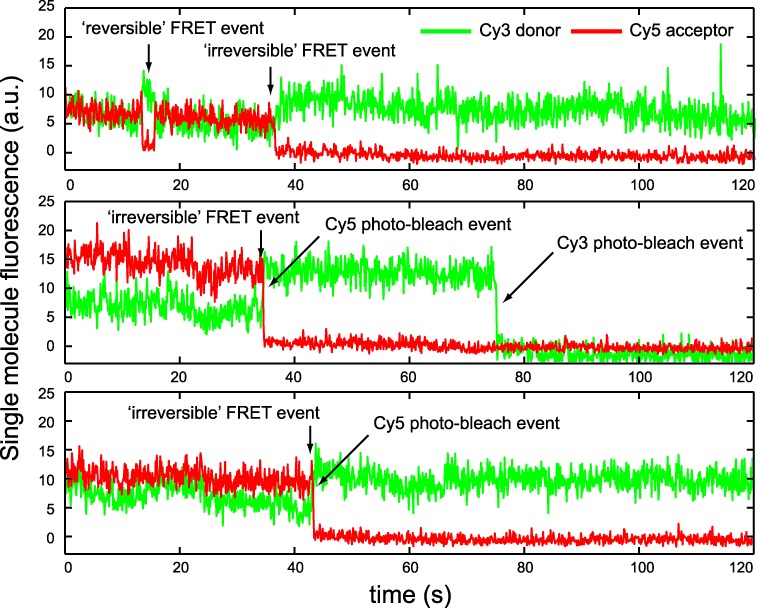
Sample smFRET trajectories for the ‘eCy3/eCy5 duplex-labeled’ DNA construct. The trajectories were recorded for ∼120 s intervals. The trajectory shown in the top panel exhibits a ‘reversible’ FRET conversion event at the 13 s tick mark. An ‘irreversible’ conversion event occurs later in the same trajectory, which is associated with Cy3 and Cy5 chromophore photo-bleaching. The middle and bottom panels show examples of trajectories that exhibit the more frequently observed photo-bleaching events.

For each of the labeled construct samples that we studied, we determined the percentage of single DNA constructs that underwent one or more FRET conversion events during a ∼120 s period while a sample area was continuously illuminated using ∼22 mW of incident laser power. In Figure 4, we show three examples of the time-dependent intensities of smFRET signals recorded from the eCy3/eCy5 duplex-labeled DNA construct. When the Cy3/Cy5 FRET pairs are strongly coupled, we expect the signal from the Cy5 acceptor chromophore to be relatively large, while the signal from the Cy3 donor chromophore will be relatively small. For the traces shown in the top panel of Figure 4, a FRET conversion event occurred near the 13 s ‘tic mark’, which was soon followed by the recovery of the FRET signal to its original value at the 15 s tic mark. Later within the same data set, a second FRET conversion event occurred (near the 37 s tick mark). Unlike the first FRET conversion event, this second event did not recover within the ∼120 s observation period.

A survey of our data suggested that whenever a reversal of a smFRET event occurred, it did so within a few seconds of the initial forward process. For the purpose of separating the two types of processes, we defined a ‘reversible’ event as one that undergoes recovery within ∼20 s of the initial process. Such reversible signal changes are thought to reflect some reasonably long-lived form(s) of local DNA ‘breathing’, a thermally driven process by which sequences of double-stranded (ds) DNA molecules transiently adopt locally ‘open’ conformations that deviate from their most stable structures and may partially expose interior single-stranded (ss) DNA template sequences (10,28–30). The middle and bottom panels of Figure 4 show examples of smFRET conversion events that were identified on this basis as ‘irreversible’—i.e. as those processes in which underlying donor and acceptor chromophore bleaching processes have occurred. DNA breathing is expected to be significantly greater near ss–ds DNA forks and p/t junctions where base-pair stacking and inter-base Watson–Crick hydrogen bonding interactions are weak (28).

**Figure 5.**
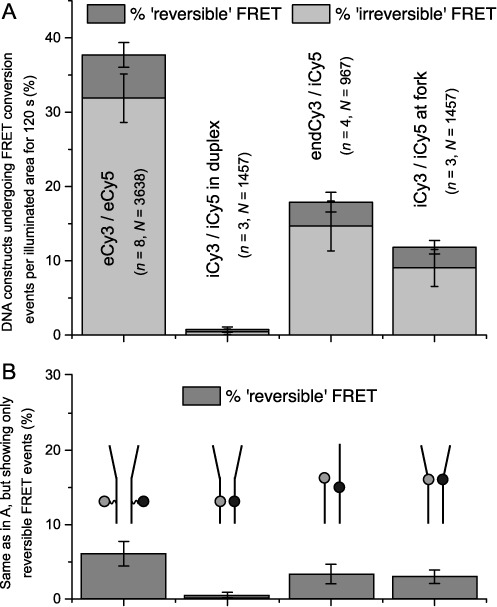
Single-molecule (sm) FRET conversion events observed for the four DNA substrates investigated. (**A**) smFRET conversion events were categorized as (reversible) fluctuations of the DNA sugar-phosphate backbone or as (irreversible) photo-bleaching of the cyanine chromophores. For each of the four substrates, the percentage of each type of process is indicated. (**B**) The percentages of reversible smFRET conversion events, attributed to DNA backbone fluctuations, are shown separately for each of the DNA substrates investigated [same data as in panel (A)].

For each of the four DNA constructs studied, we determined the relative populations of reversible versus irreversible smFRET conversion events during a 120 s observation period of given sample areas (Figure 5 and Table 1). We found that for eCy3/eCy5 duplex-labeled DNA constructs, ∼38% of the molecules within the imaging area exhibited FRET conversion activity, which was partitioned into ∼6% reversible events and ∼32% irreversible events on the basis of the time criteria listed above. In contrast, for the iCy3/iCy5 duplex-labeled DNA construct, we found that total FRET conversion activity was nearly undetectable (<1%). The endCy3/iCy5 p/t junction-labeled DNA construct exhibited a mid-range activity of FRET conversion of ∼18%, with ∼3% reversible conversions and ∼15% irreversible conversions. We observed a slightly lower level of conversion activity for the iCy3/iCy5 fork-labeled DNA construct ∼12%, with ∼3% reversible conversions and ∼9% irreversible.

We note that the pattern of FRET conversion activity (both reversible and irreversible) for the four different samples closely tracked that of the photo-bleach lifetimes for the four DNA constructs obtained in our previous measurements (Figure 3A and Supplementary Figure S3). Moreover, for all of the samples we investigated, the subpopulation of reversible FRET conversion events was a minor fraction of the total FRET activity. As stated above, we associate reversible FRET conversion events with spontaneous relatively long-lived conformational changes of the sugar-phosphate backbone, and as shown in Figure 5, such backbone fluctuations are much less likely to occur for DNA constructs in which the chromophore probes are rigidly inserted into the sugar-phosphate backbone of the duplex, in comparison to the three remaining constructs in which the probes are relatively exposed to the solvent and less rigidly positioned to varying degrees. The DNA constructs that utilized external labeling chemistry appeared to be exceptionally unstable, exhibiting a ∼20-fold greater likelihood of undergoing conformational fluctuations within the duplex region than DNA constructs that employ internal probes. DNA constructs labeled at the p/t and replication fork junctions again appeared to undergo mid-range levels of backbone conformation fluctuations.

The results summarized in Figure 5 and detailed in Table 1 were based on 3–8 separate experiments for each construct (*n*), as shown, and involved the analysis of 1000–4000 individual FRET conversion events (*N*) for each construct. The error bars for each set of results are shown. The total FRET conversion results are summarized in Figure 5A, and the reversible FRET conversion events are also shown separately in Figure 5B to emphasize the major differences between the constructs with respect to this parameter. Again, comparison of the heights of the first and second columns in these bar graphs emphasizes the enormous decrease in both reversible and irreversible spontaneous FRET conversions brought about by moving the dye chromophores from ‘flexible external’ to ‘rigid internal’ labeling positions.

**Figure 6.**
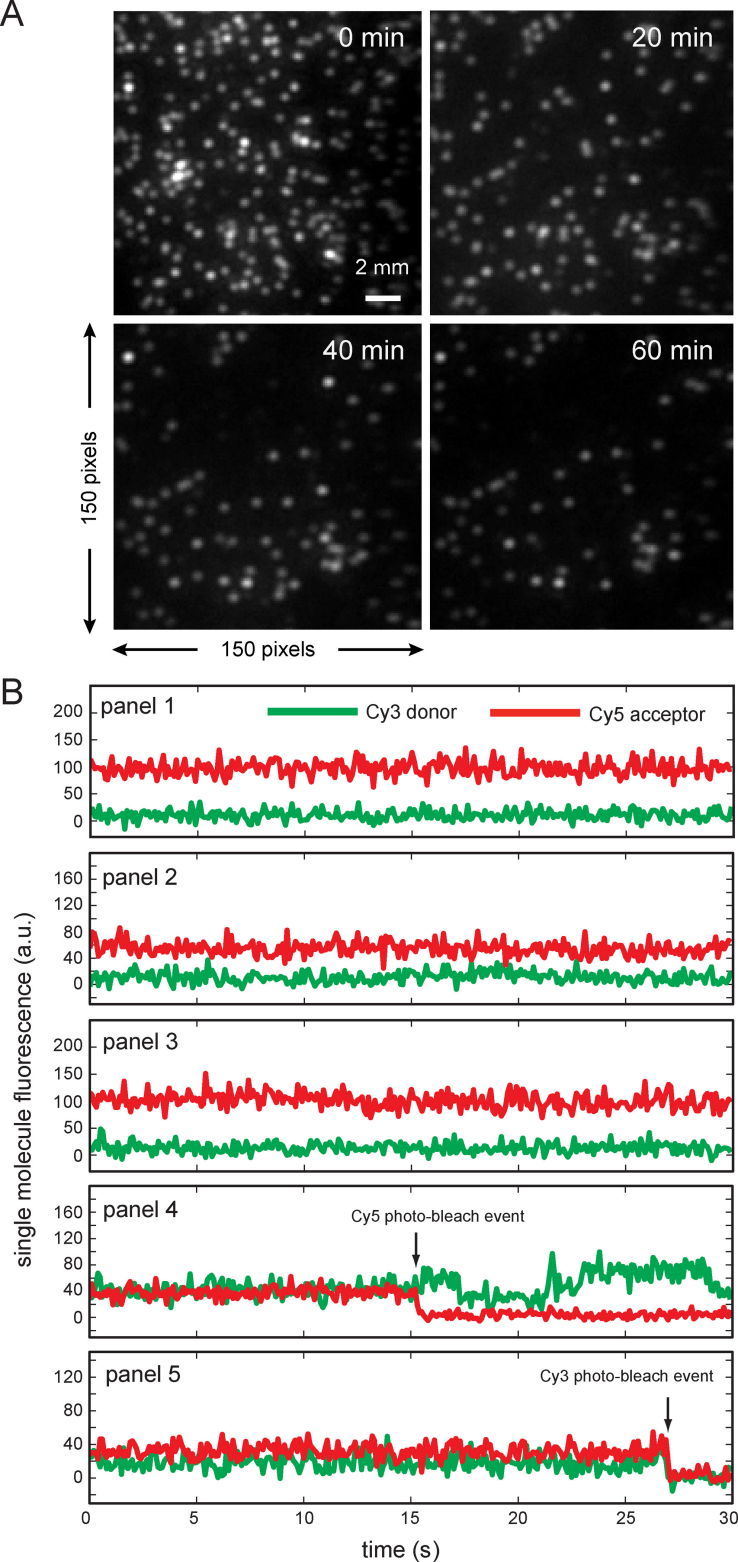
Fluorescence images of the Cy5 detection area for the ‘iCy3/iCy5 duplex-labeled’ DNA constructs and representative Cy3 and Cy5 sm trajectories. (**A**) Fluorescence images of the Cy5 detection area for the ‘iCy3/iCy5 duplex-labeled’ DNA constructs. Images were obtained at 20-min intervals under continuous laser excitation (∼10 mW, see Supplementary Figure S8). (**B**) Representative Cy3 and Cy5 sm trajectories recorded from ‘iCy3/iCy5 duplex-labeled’ DNA constructs over a 30-s interval after ∼60 min of laser excitation (∼10 mW). After ∼60 min of continuous laser excitation, ∼33% of the FRET pairs remained stable and non-blinking for both Cy3/Cy5 donor–acceptor FRET pairs (as shown in panels 1, 2 and 3). The infrequent occurrence of Cy3 and Cy5 photo-bleaching events could also be observed during the same data acquisition period (as shown in panels 4 and 5).

In Figure 6, we present a typical data set for an internally labeled construct continuously irradiated at 10 mW for 1 h. These data illustrate the overall long-term stability of the entire collection of labeled constructs (Figure 6A). We further note the very infrequent occurrence of either Cy3 or Cy5 photo-bleaching events, as well as the virtual absence of reversible (‘breathing’) FRET conversion events for these internally labeled constructs (panels 1 through 5 of Figure 6B).

### Effects of labeling procedures on the local thermodynamic stability of the DNA constructs

In order to compare these results with the effects of internal versus external labeling on the *structural stability* of the local DNA duplexes, we also examined the effects of dye placement and geometry on the cooperative melting temperatures of the duplex DNA portions of the various constructs at a number of salt concentrations, and compared the results with expectations based on duplex composition, length and sequence. The results are summarized in Table 2 and show—contrary to our initial expectations—that under all the salt concentration conditions tested the *internally labeled* dye pairs lowered the overall stability of the duplex portions of our constructs significantly *less* than did the *externally labeled* pairs. Table 2 shows that our DNA constructs with external dye probes located well within the duplex regions at three different salt concentrations were destabilized by an average of ∼ −6.5°C, while the introduction of pairs of internally labeled dyes into the same positions of the DNA duplex resulted in average melting temperature decreases of only ∼ −2.7°C.

**Table 2.** Comparison between experimental and ‘theoretical’ melting temperature (*T*_m_) values of DNA replication fork constructs and the effects chromophore insertion site position, chemistry and buffer conditions on *T*_m_

DNA constructs	Experimental *T*_m_ (°C)	Theoretical *T*_m_ (°C)^e^
Unmodified DNA^a^	69.6	69.6
Unmodified DNA^b^	68.5	67.6
Unmodified DNA^c^	68.4	65.8
iCy3/iCy5-labeled DNA^a,d^	66.8	68.9
iCy3/iCy5-labeled DNA^b,d^	65.8	67.0
iCy3/iCy5-labeled DNA^c,d^	65.8	65.3
eCy3/eCy5-labeled DNA^a^	63.1	70.0
eCy3/eCy5-labeled DNA^b^	62.0	68.3
eCy3/eCy5-labeled DNA^c^	61.9	66.8

^a^Measured in 200 mM NaCl, 12 mM MgCl_2_, 10 mM Tris (pH 8.0).

^b^Measured in 100 mM NaCl, 6 mM MgCl_2_, 10 mM Tris (pH 8.0).

^c^Measured in 50 mM NaCl, 3 mM MgCl_2_, 10 mM Tris (pH 8.0).

^d^For theoretical *T*_m_ calculation, a mismatched base pair (dA–dG) was placed at the location of the Cy3/Cy5 probe.

^e^Melting temperatures were predicted using unified nearest-neighbor parameters provided by the DINAMelt server, which are based on hybridization calculations for two separate DNA strands (35).

Calculations summarized in Table 2 also showed that the destabilization introduced by the internally labeled dye pair was close to that expected for our duplex sequence by the introduction of a single-unmatched base pair at the position of the Cy3/Cy5 pair, while the destabilization introduced by the external labels was much larger, even though the externally labeled constructs contained *no* mismatched base pairs (see Supplementary Table S1 for DNA sequences).

## DISCUSSION

We have demonstrated that the photo-stability of Cy3/Cy5-labeled DNA constructs is significantly influenced by labeling chemistry and dye insertion site position. Our results are consistent with the suggestion that the photo-stability of these cyanine dyes may be enhanced by rigidly incorporating the chromophores within the duplex region, perhaps because increased structural rigidity of the dye environment may help to decouple the electronic transitions of the chromophores from local vibrational motions. Our results also suggest that photo-bleaching of the chromophore probes is more likely to occur at DNA construct sites at which long-lived breathing fluctuations (‘reversible’ FRET conversion events) are most pronounced, thus decreasing the rigidity of the chromophores and also increasing their exposure to the solvent environment. Consistent with this pattern, we have found that the externally labeled duplex system (Figure 1A) is the least photo-stable of the four DNA constructs we examined, while in contrast we observed dramatically *enhanced* photo-stability of internally labeled duplex DNA constructs. Thus, even after ∼1 hr of continuous excitation at ∼10 mW of incident laser power, ∼33% of the originally detected iCy3/iCy5 smFRET pairs still exhibited stable fluorescence signals (see Figures 3 and 6 and Supplementary figure S8).

Moreover, we have also shown that the thermodynamic stability of the externally labeled DNA duplex constructs is significantly reduced in comparison to that of internally labeled and unlabeled constructs (Table 2), while the replacement of a base pair by the Cy3/Cy5 pair in the internally labeled construct (comprising the equivalent of a base-pair mismatch) might lead us to expect the opposite. We speculate that this thermodynamic destabilizing effect of the external labeling chemistry might reflect transient destabilizing interactions between the cyanine residues and the natural base pairs, perhaps as a consequence of partial and transient intercalation reactions that could locally disrupt the normal base stacking and Watson–Crick (W-C) base-pair interactions. Such fluctuations might also be expected to result in the long-lived reversible FRET-conversion events we observed with the external labels, and the flexibility of the tether connecting the chromophores to the DNA backbones may accentuate these destabilizing effects by permitting a broad range of such interactions due to random fluctuations of the tether. This is consistent with the lack of a defined CD spectrum in the dye absorbance region of the external probes, showing that such dye pairs (in contrast to internally labeled pairs) do not form stable chiral structures.

There is precedent for such transient interactions of external dyes affecting the structural stability (as measured by Δ*T*_m_ changes) of vicinal duplex DNA (31–34). For example, in an earlier study Iqbal *et al.* (34) showed that Cy3 and Cy5 dyes located at the *ends* of DNA duplex constructs may stack on the ends of the duplex, in that case resulting in an *increase* in the thermodynamic stability of the construct as well as restricting the relative orientations of the FRET dye pair. Our finding in the present study, which is also consistent with earlier work (33), shows that transient partial local dye binding and/or intercalation of *external* probes located deeper in the duplex can instead be thermodynamically *destabilizing*, perhaps by interacting with and stabilizing unfavorable fluctuations and bending events. In contrast, as we show in this paper, *internal* probes within the duplex do not significantly alter the local stability of the construct [beyond what is expected of a non-complementary base pair (35)].

**Figure 7.**
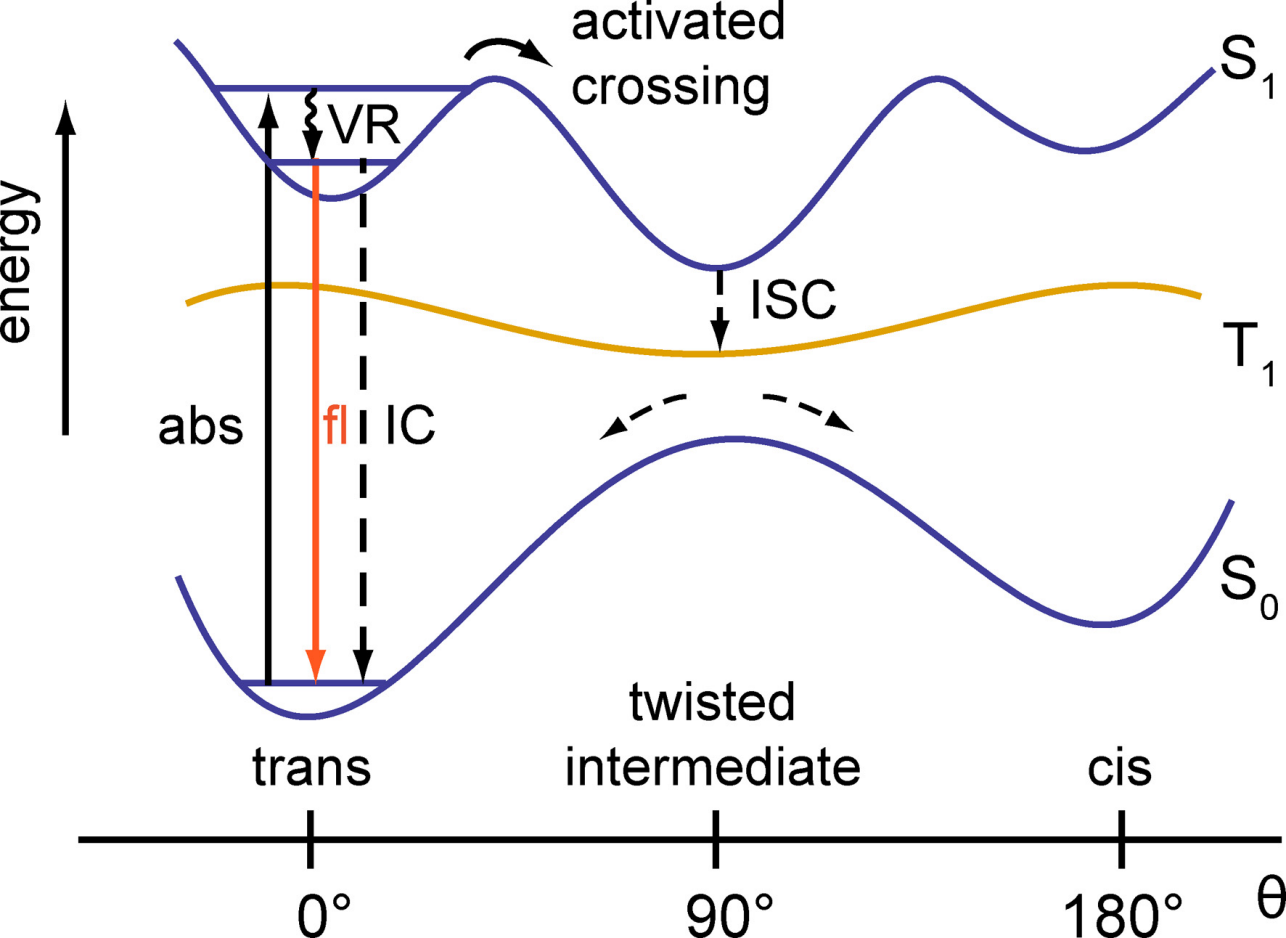
Hypothetical electronic potential energy surfaces are shown as a function of the coordinate *θ*, which specifies a C–C bond rotation within the polymethine chain of the cyanine chromophore. The ground state surface is labeled S_0_, the excited singlet state is labeled S_1_ and the excited triplet state is labeled T_1_. Absorption by the trans conformation (*θ* = 0°) creates a vibrationally excited state in S_1_, which can undergo efficient vibrational relaxation (VR) followed by IC or fluorescence. Alternatively, the molecule may undergo vibrationally activated photo-isomerization to the more stable ‘twisted intermediate’ (*θ* = 90°), which can undergo branching to produce ground state population in both cis (*θ* = 180°) and trans conformations. Intersystem crossing (ISC) to the triplet state T_1_ is favored by the formation of the twisted intermediate. The twisted T_1_ state is the primary intermediate through which photo-degradation of the cyanine chromophore occurs.

It is possible to explain the origins of the enhanced photo-stability we have observed for internally labeled Cy3/Cy5 DNA constructs in mechanistic terms. A great deal of information is available about the photo-physics of free cyanine dyes in solution, and there is evidence that steric hindrance of the chromophore when attached to a biological macromolecule can affect its fluorescence intensity (19–20). This effect can be understood as a consequence of photo-isomerization of the molecule, in keeping with the scheme shown in Figure 7. The polymethine chains of both chromophores exist primarily in the energetically favorable ‘all-trans’ conformation. This trans conformation can undergo absorption of light, inducing a vertical (Franck–Condon) transition from the electronic ground state S_0_ to a vibrationally active first excited singlet state S_1_. Relaxation of the initially excited trans conformation can occur through a number of different pathways. In one case, excess vibrational energy is rapidly dissipated as heat to the surroundings, producing amplitude on the vibrationally unexcited S_1_ surface, which may then undergo radiationless internal conversion (IC) and fluorescence on sub-nanosecond time scales. Alternatively, the molecule in its electronic-vibrationally excited state has little C = C double-bond character, and it may thus undergo rotation about one of its C–C bonds to form the sterically favorable ‘twisted intermediate’.

Figure 7 illustrates the (hypothetical) potential energy surfaces that govern these processes. The photo-isomerization is depicted as an activated barrier crossing in which the excess vibrational energy is coupled to the C–C bond rotation. The existence of the twisted intermediate provides a route in which ground state populations in both cis and trans conformations can be generated through radiationless relaxation processes. Because the S_1_ ← S_0_ transition in the cis conformation carries little oscillator strength, the molecule in the cis conformation will appear non-fluorescent until it is restored to its all-trans conformation through thermally activated re-equilibration.

Previous work on the photo-stability of cyanine-labeled DNA has focused on the role of long-lived excited triplet (T) states, which can irreversibly react with ground-state oxygen to form non-fluorescent photoproducts (13,18,36). Similar to other systems that undergo photo-isomerization, there must exist an excited triplet state (T_1_) surface lower in energy relative to that of the S_1_ surface, as depicted in Figure 7 (37). Just as for the S_1_ state, the T_1_ state should also be stabilized by the twisted intermediate conformation. Thus, intersystem crossing (ISC) from the S_1_ surface to the T_1_ surface should be coupled to the photo-isomerization, with the most stable (and longest lived) form of the triplet corresponding to the twisted intermediate. We hypothesize that this twisted T_1_ species is the primary intermediate by which photo-degradation occurs in cyanine-labeled DNA constructs.

The above model provides a plausible framework to understand our experimental observations. Photo-isomerization facilitated photo-degradation is least likely to occur for the ‘iCy3/iCy5 duplex-labeled’ DNA construct due to the steric constraints placed on the distal ends of the chromophores, which are rigidly positioned within the structurally ordered duplex. These steric constraints should be directly correlated to the degree of order at the chromophore insertion site position, which can be associated with the barrier height for the photo-isomerization process. Therefore, consistent with our observations, we can predict the following progression of decreasing steric hindrance and correspondingly decreasing photo-stability: ‘iCy3/iCy5 duplex-labeled’ > ‘iCy3/iCy5 fork-labeled’ DNA construct >> ‘endCy3/iCy5 p/t-labeled’ DNA construct > ‘eCy3/eCy5 duplex-labeled’ DNA construct.

Clearly, the model we have proposed will require experimental verification. However, we note that our principal results suggest an alternative labeling strategy for single-molecule ‘substrates’ that departs qualitatively from most of the previously implemented approaches for such research. Specifically, we anticipate that these findings may lead to new ways to extend the observation time windows for single-molecule fluorescence experiments in general and perhaps also minimize local stability changes in DNA duplex structure that may be induced by less rigidly inserted probes.

## SUPPLEMENTARY DATA

Supplementary Data are available at NAR Online.

SUPPLEMENTARY DATA
