# Supplementary material for: Internally labeled Cy3/Cy5 DNA constructs show greatly enhanced photo-stability in single-molecule FRET experiments
Source: Nucleic Acids Res. 2014 Mar 15;42(9):5967–77. doi: 10.1093/nar/gku199 (PMC4027219; doi:10.1093/nar/gku199)
Supplement: SUPPLEMENTARY DATA [file supp_42_9_5967__index.html]

Internally labeled Cy3/Cy5 DNA constructs show greatly enhanced photo-stability in single-molecule FRET experiments — Internally labeled Cy3/Cy5 DNA constructs show greatly enhanced photo-stability in single-molecule FRET experiments — SUPPLEMENTARY DATA 

# Internally labeled Cy3/Cy5 DNA constructs show greatly enhanced photo-stability in single-molecule FRET experiments

## SUPPLEMENTARY DATA

**Files in this Data Supplement:**

- Supplemental Figures
