# Supplementary material for: Internally labeled Cy3/Cy5 DNA constructs show greatly enhanced photo-stability in single-molecule FRET experiments
Source: Nucleic Acids Res. 2014 Mar 15;42(9):5967–77. doi: 10.1093/nar/gku199 (PMC4027219; doi:10.1093/nar/gku199)
Supplement: SUPPLEMENTARY DATA [file supp_gku199_nar-03554-h-2013-File003.pdf]

Supplementary Data: Internally-labeled Cy3 / Cy5 DNA constructs show greatly enhanced photo-stability in single-molecule FRET experiments

Wonbae Lee[1,2], Peter H. von Hippel[2,*], and Andrew H. Marcus[1,2,*]

[1.] Oregon Center for Optics and Department of Chemistry, University of Oregon, Eugene, OR 97403

[2.] Institute of Molecular Biology and Department of Chemistry, University of Oregon, Eugene, OR 97403

[*] Corresponding authors: petevh@molbio.uoregon.edu, ahmarcus@uoregon.edu

Fluorescently labeled DNA replication fork constructs, and the effects of labeling chemistry and insertion site position on thermodynamic stability

Table S1. ***Nucleotide base sequences and nomenclature for the DNA constructs used in these studies*.***

DNA construct	Nucleotide base sequence for smFRET experiments
eCy3/eCy5 duplex-labeled	3′-TCTCCTCGTCTCCCTCGTGTCGTCTCGTCAGTATTATACGC3ACGCTAATATACCACG-dT_{29}-5′<br>5′-**Biotin**-AGAGGAGCAGAGGGAGCACAGCAGAGCAGTCATAATATGCGA5GCGATTATATATGCTTTTACCACTTTCACTCACGTGCTTAC-3′
iCy3/iCy5 duplex-labeled	3′-GTCAGTATTATACGCTiCy3CGCTAATATACCACG-dT_{29}-5′<br>5′-**Biotin**-CAGTCATAATATGCGAiCy5GCGATTATATATGCTTTTACCACTTTCACTCACGTGCTTAC 3′
endCy3/iCy5	5′-GCAAGAACCGAACCAA-Cy3-3′<br>3′-**Biotin**-CGTTCTTGGCTTGGTiCy5TGGACTAATC-5′
iCy3/iCy5 fork-labeled	5′-CTCCCTCGTGTCGTCTCCAGTCATAATATGCGAiCy3ATGCTTTTACCACTTTCACTCACGTGCTTA-3′<br>3′-**Biotin**-AGAGGAGCAGAGGGAGCACAGCAGAGGTCAGTATTATACGCTiCy5CGCTAATATACCACG-dT_{29}-5′
**DNA construct**	**Nucleotide base sequence for CD and thermal denaturation measurements**
Unmodified duplex-labeled	3′-GTCAGTATTATACGCTCGCTAATATACCACGTTTTTTTTTTTTTTTTTTTTTTTTTTTTT-5′<br>5′-**Biotin**-CAGTCATAATATGCGAGCGATTATATATGCTTTTACCACTTTCACTCACGTGCTTAC-3'
iCy3/iCy5 duplex-labeled	3′-GTCAGTATTATACGCTiCy3CGCTAATATACCACG-dT_{29}-5′<br>5′-**Biotin**-CAGTCATAATATGCGAiCy5GCGATTATATATGCTTTTACCACTTTCACTCACGTGCTTAC-3'
eCy3/eCy5 duplex-labeled	3′-GTCAGTATTATACGC3ACGCTAATATACCACG-dT_{29}-5′<br>5′-**Biotin**-AGAGGAGCAGAGGGAGCACAGCAGAGCAGTCATAATATGCGA5GCGATTATATATGCTTTTACCACTTTCACTCACGTGCTTAC-3′

* eCy3 refers to the 'external' labeling chemistry of Cy3 via the amino-modified C6-position of dT, eCy5 refers to the 'external' labeling of Cy5 via the amino-modified C6-position of dT; 3 represents the amino modified C6-position labeled dT (labeled with Cy3); 5 represents the amino modified C6-position labeled dT (labeled with Cy5); iCy3 refers to the 'internal' phosphoramidite-based labeling chemistry used to insert Cy3 into the sugar-phosphate backbone; iCy5 refers to the 'internal' phosphoramidite-based labeling chemistry used to insert Cy5 into the sugar-phosphate backbone; endCy3 refers to end-labeled position generated through phosphoramidite chemistry. For all three of the DNA constructs shown, underlines represent the double-stranded regions. Biotin-neutravidin linkages were used to attach the constructs to the surface of a glass coverslip. The probe insertion chemistries used are shown in Figure S1.

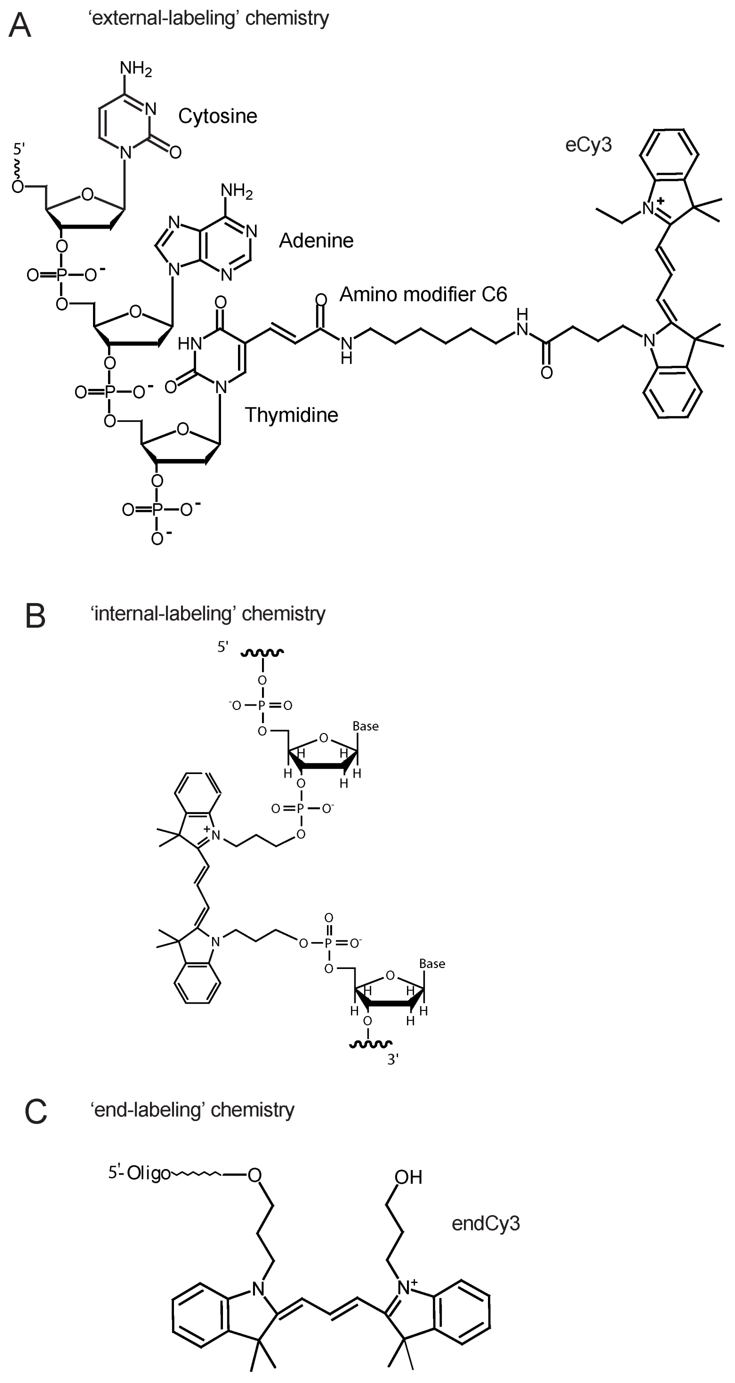

Figure S1. ***Labeling strategies used to attach the Cy3 and Cy5 FRET chromophores, showing the insertion chemistry used to label the DNA replication fork constructs.*** (**A**) 'External-labeling' chemistry. (**B**) 'Internal-labeling' chemistry. (**C**) 'End-labeling' chemistry.

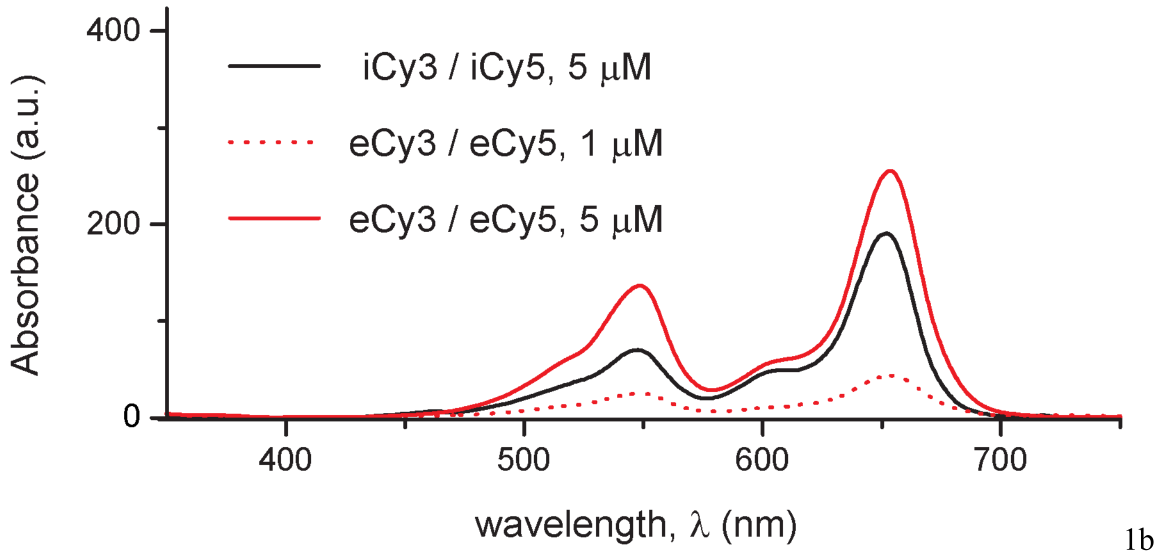

1b

Figure S2. ***The absorbance spectra of the internally and externally labeled Cy3 / Cy5 dsDNA constructs are very similar, with the Cy3 and Cy5 absorption maxima occurring at ~549 nm and ~649 nm, respectively.***

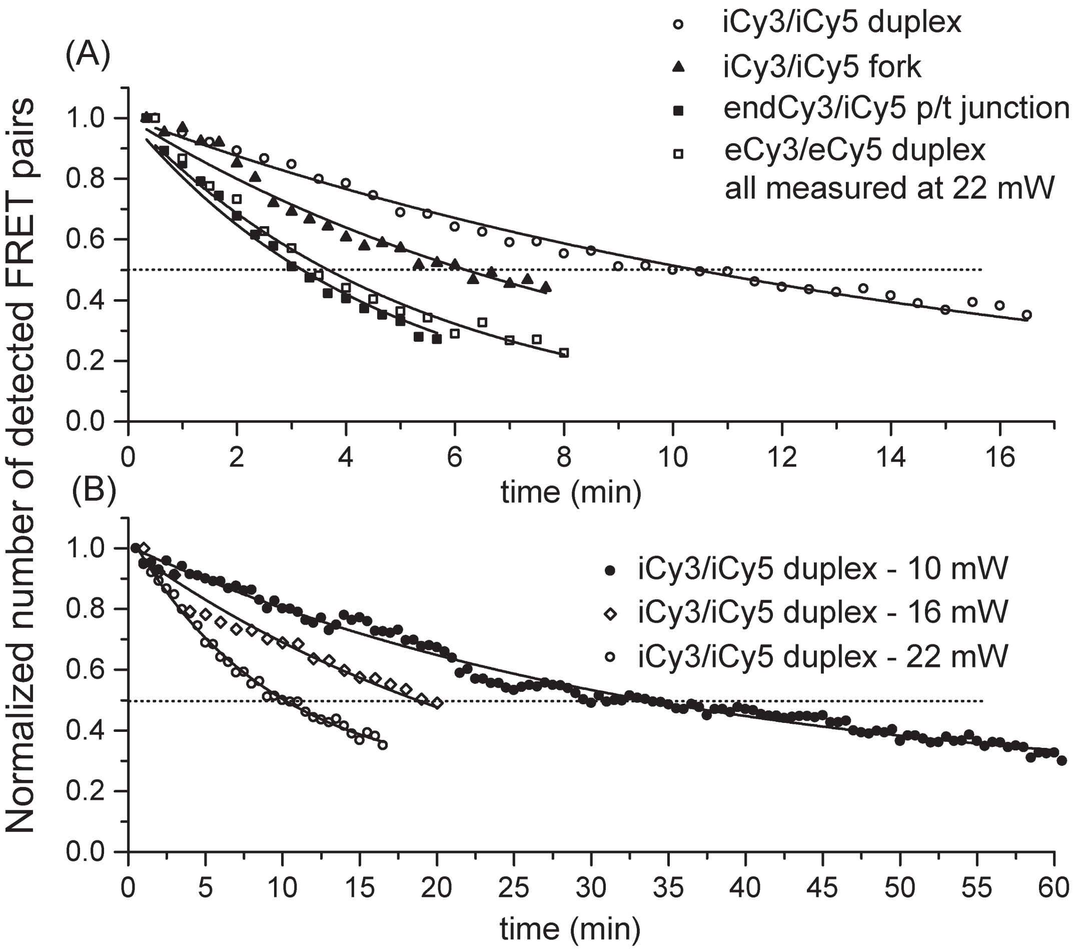

Figure S3. ***Single exponential decay plots of the number of Cy3 and Cy5 FRET chromophore pairs detected within a fixed imaging area as a function of time of continuous illumination at 532 nm.*** The total number of detected FRET pairs ranged from 308 to 542. (**A**) The laser power was set to ~22 mW. All four of the DNA substrates exhibited an exponential time-course of photo-bleaching, with rates depending on chromophore position and insertion chemistry (see Figures 1A-D, 3A, and S4-6). (**B**) The laser power dependence of the photo-bleach rate for the iCy3 / iCy5 duplex-labeled DNA construct (see Figures 1B, 3B, S7 and S8).

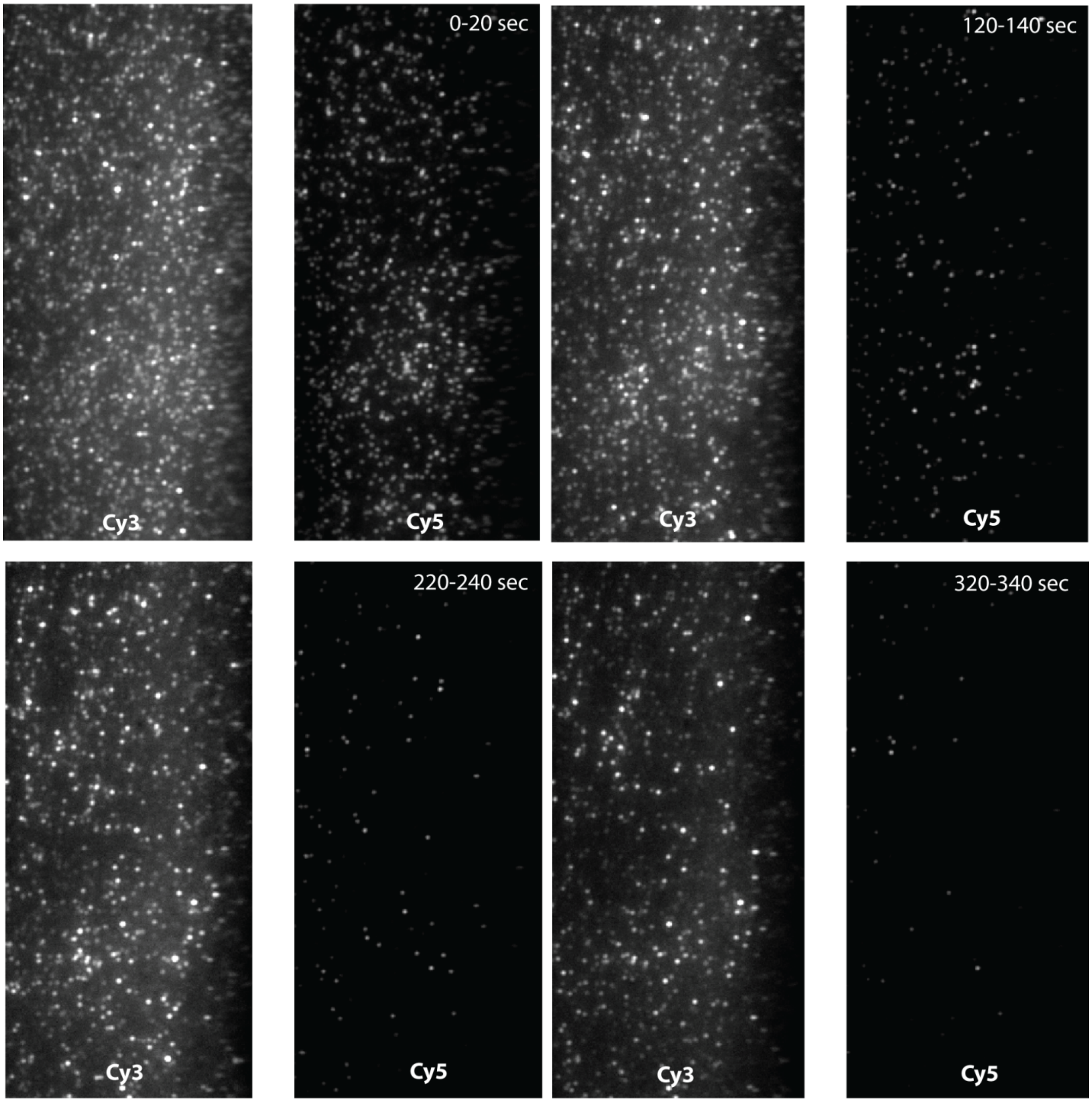

Figure S4. ***Sample images obtained with the 'eCy3/eCy5' dsDNA construct.*** Images were recorded at different times during the continuous laser excitation (~22 mW at 532nm) as specified. The image is split into donor (left) and acceptor (right) channels, each 30 µm x 60 µm. A donor molecule on the left and the corresponding acceptor molecule are horizontally displaced from one another by 255 pixels.

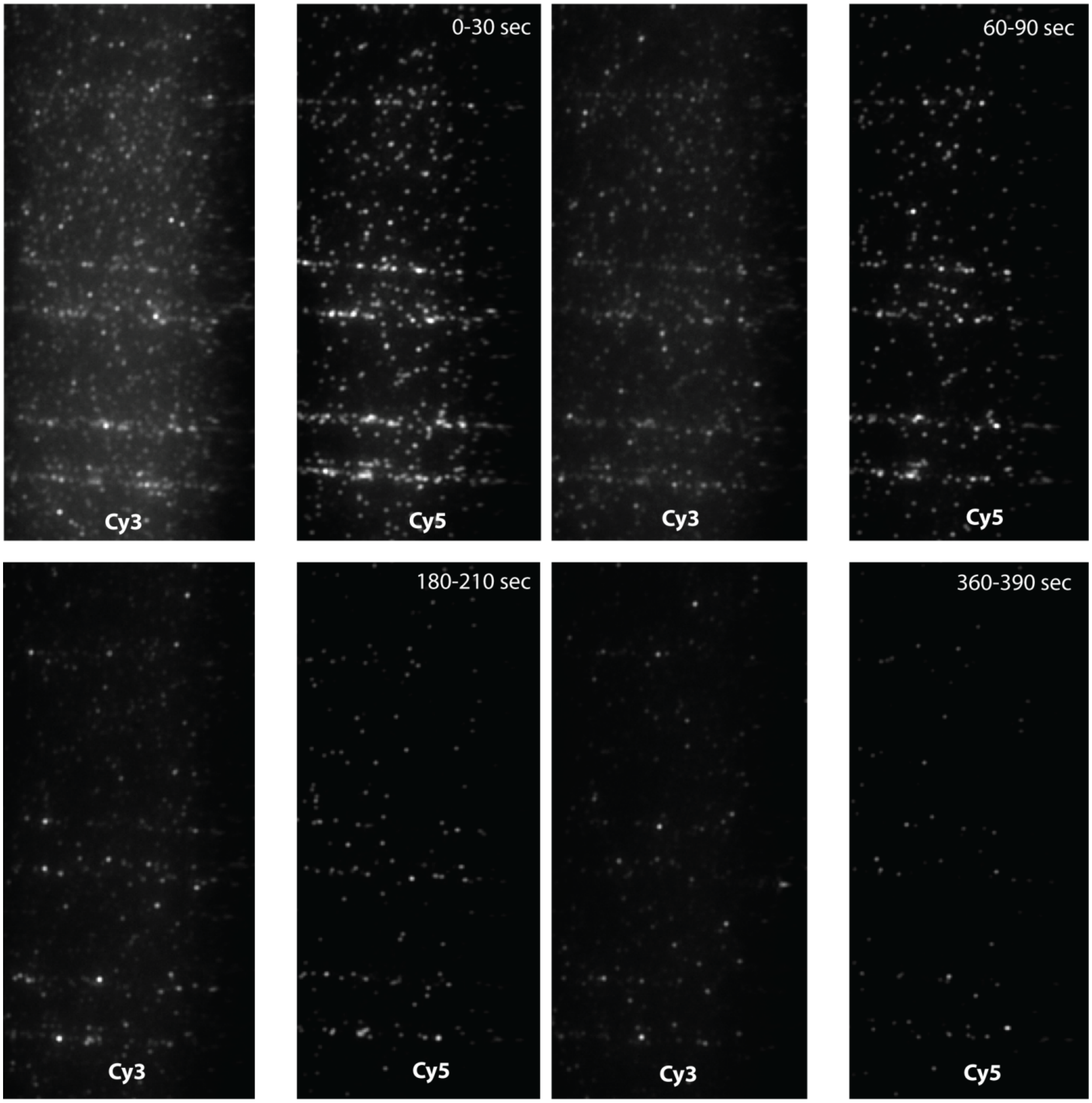

Figure S5. ***Sample images obtained with the 'endCy3 / iCy5' labeled p/t DNA construct.*** Images were recorded at different times during continuous laser excitation (~22 mW at 532nm), as specified.

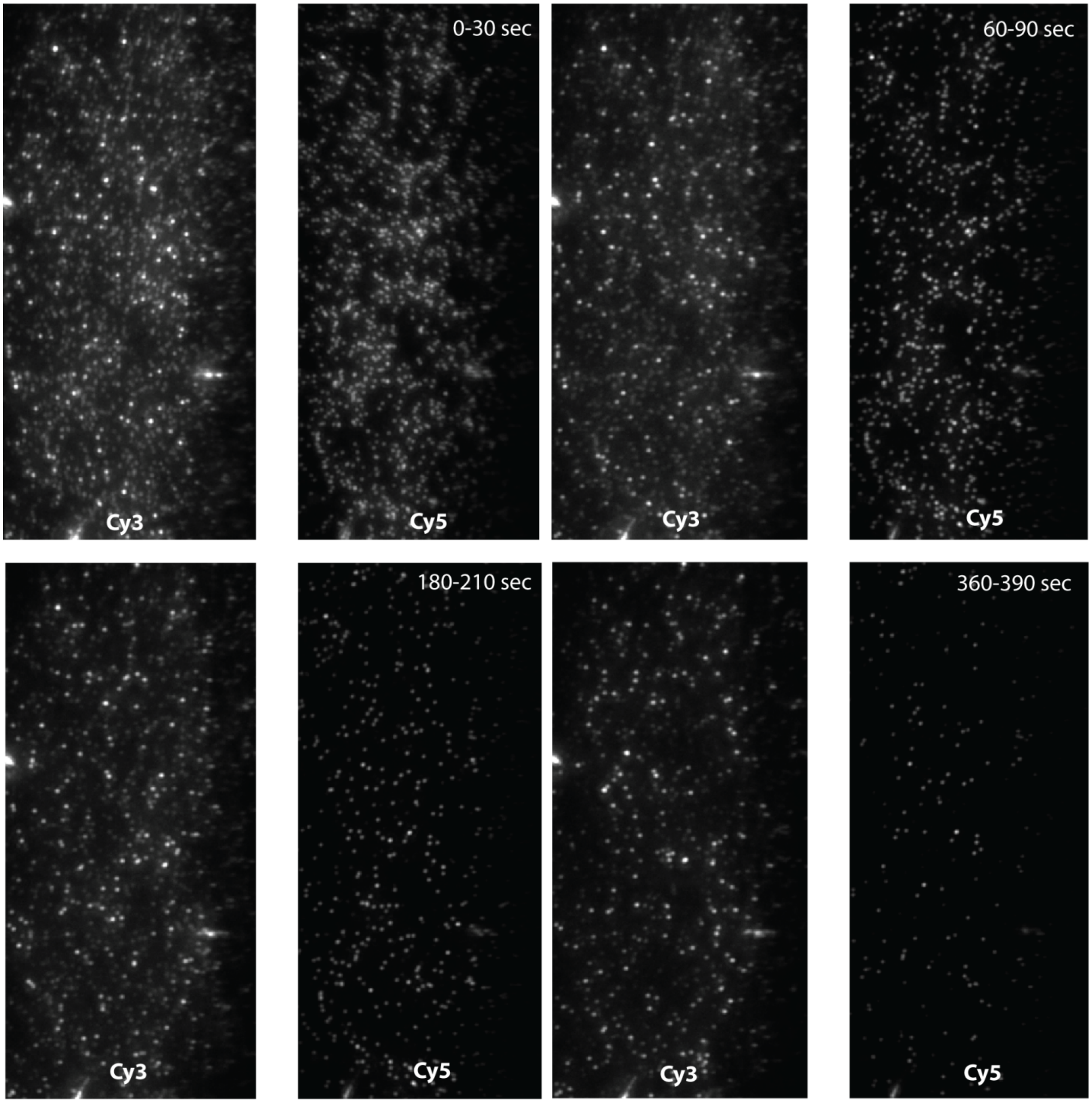

Figure S6. ***Sample images obtained with the 'iCy3 / iCy5' fork-labeled DNA construct.*** Images were recorded at different times during continuous laser excitation (~22 mW at 532nm), as specified.

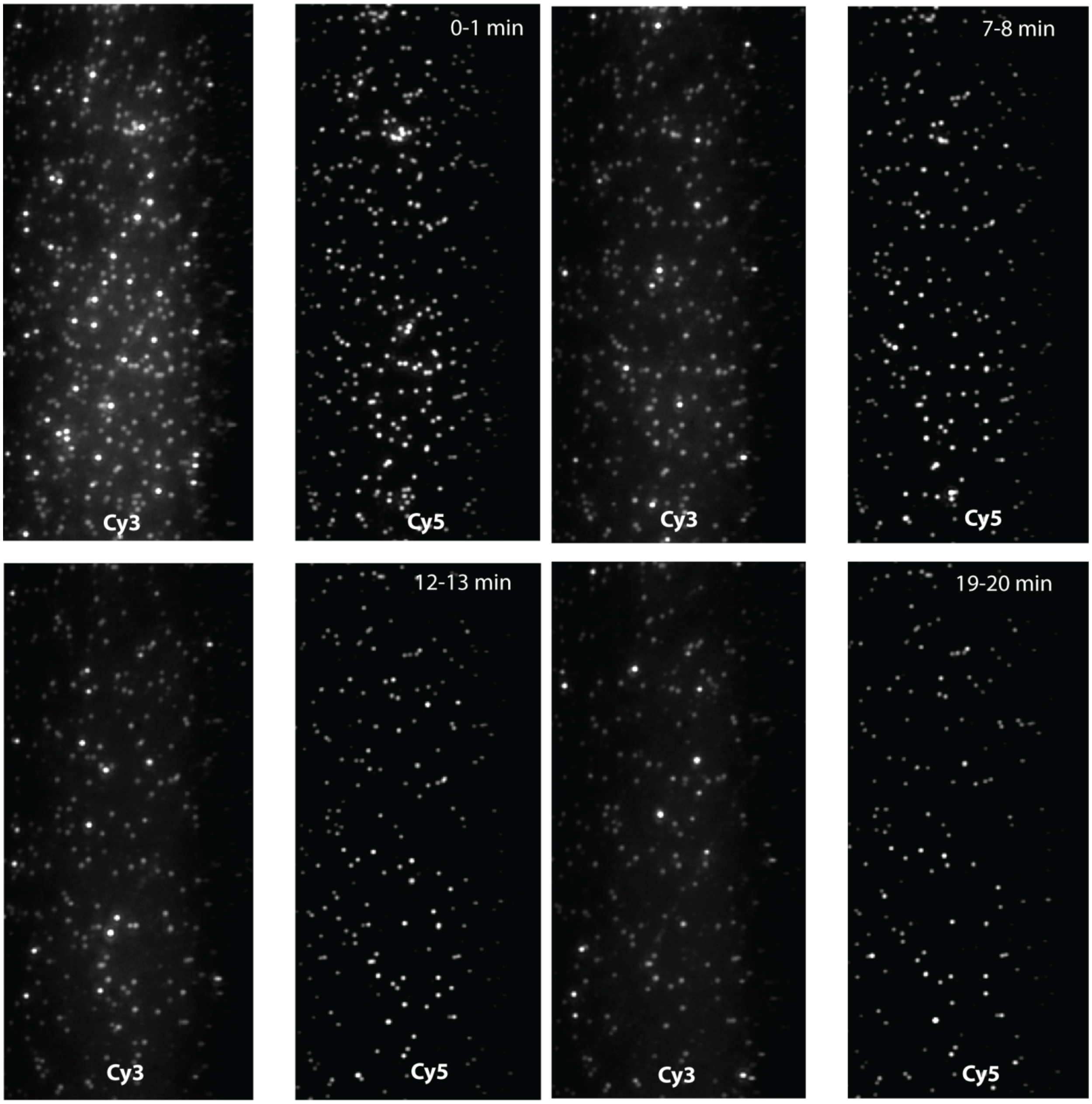

Figure S7. ***Sample images obtained with the 'iCy3 / iCy5 duplex-labeled' DNA construct.*** Images were recorded at different times during continuous laser excitation (~22 mW at 532nm), as specified.

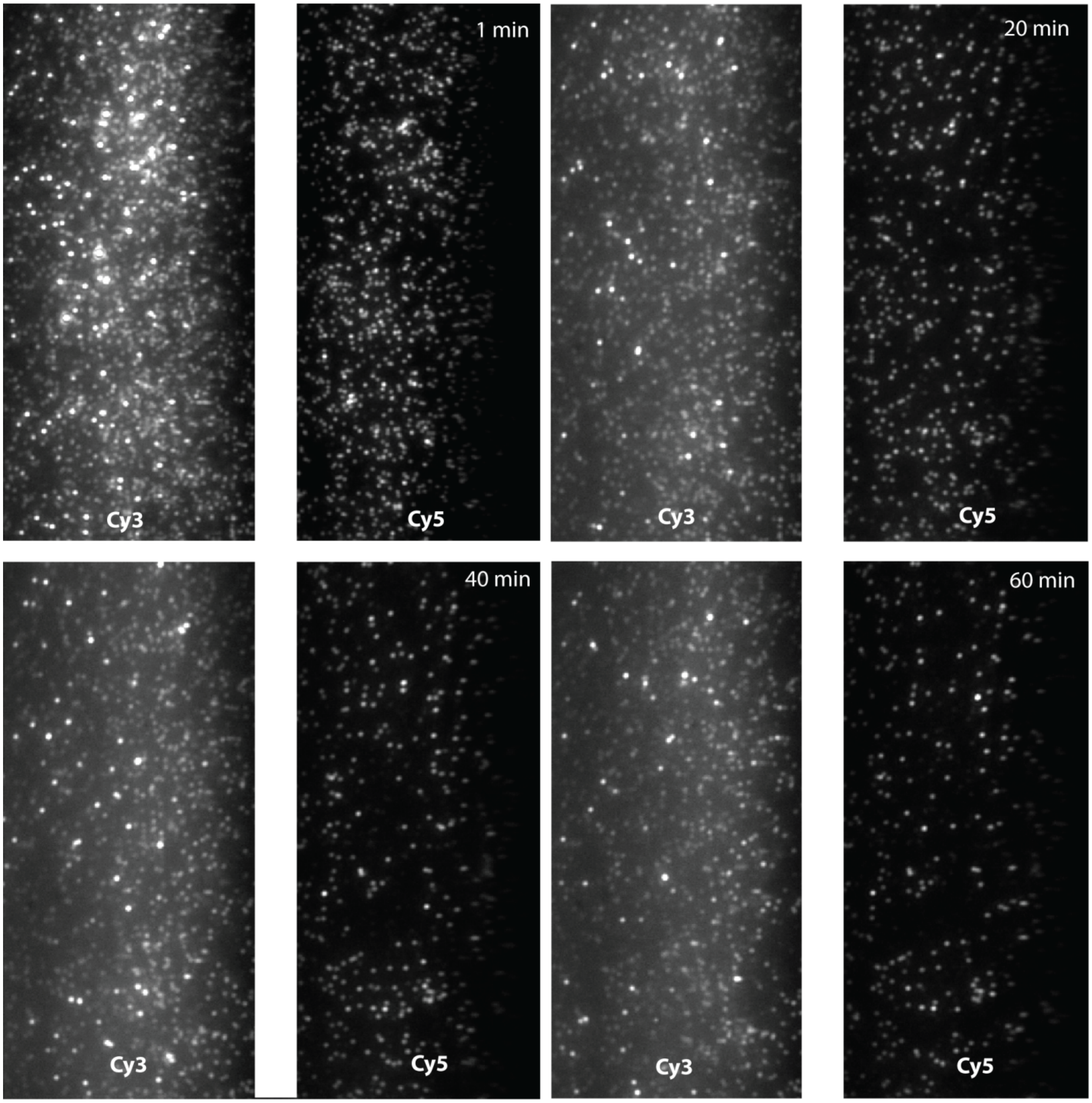

Figure S8. ***Sample images obtained with the 'iCy3 / iCy5 duplex-labeled' DNA construct.*** Images were recorded at different times during continuous 532 nm laser excitation at *~10mW*, as specified. After ~60 minutes of continuous laser excitation at 10 mW, ~33% of the FRET pairs remained stable and non-blinking.
